# Efficacy and safety of trastuzumab deruxtecan for metastatic HER2+ and HER2-low breast cancer: A systematic review and meta-analysis

**DOI:** 10.1097/MD.0000000000045936

**Published:** 2025-11-14

**Authors:** Yujing Mu, Jianrong Li, Yingyi Fan, Xiaohua Pei

**Affiliations:** a Department of Breast Surgery, Third Afiliated Hospita of Beijing University of Chinese Medicine, Beijing, China.

**Keywords:** breast cancer, human epidermal growth factor receptor 2, meta-analysis, trastuzumab deruxtecan

## Abstract

**Background:**

Trastuzumab deruxtecan (T-DXd) is a novel antibody-drug conjugate uesd for the treatment of HER2- positive (HER2+)breast cancer. This systematic review aimed to evaluate the efficacy and safety of T-DXd in advanced HER2-positive breast cancer.

**Methods:**

PubMed, Web of Science and Embase databases were searched for literature on trastuzumab deruxtecan treatment for HER2-positive or low-expression breast cancer before December 30, 2024. The outcome measures were progression-free survival (PFS), overall survival (OS), objective response rates, and adverse events of any grade and grade ≥ 3. Meta-analysis of the relevant data was performed using Stata 14.0.

**Results:**

A total of 2995 patients from 7 studies were included. The median PFS and median overall survival of patients in the T-DXd group were significantly longer than those of patients in the control group (mPFS HR = 0.43;95% CI:0.31–0.62, p<0.05; mOS HR = 0.72; 95% CI:0.64–0.82, p<0.05). Further subgroup analyses based on differences in hormone receptor expression, occurrence of brain metastases, visceral basal conditions, and previous lines of treatment showed that all patients in the T-DXd group had significantly longer mPFS than those in the control group (*P* < .05). The objective remission rate of patients in the T-DXd group was significantly higher than that of patients in the control group (RR = 2.31; 95%CI: 1.88–2.85, P<0.05). In addition, the T-DXd also increased the incidence of adverse events such as anemia, nausea, vomiting, constipation and interstitial lung disease, but the incidence of neutropenia, diarrhea, and alopecia was not significantly different between the 2 groups. No significant publication bias was observed in this study. The results of the sensitivity analysis showed high heterogeneity in mPFS, but the results obtained after excluding the DESTINY-Breast04 studies were all more robust.

**Conclusion:**

T-DXd has significant long-term and near-term efficacy in prolonging median overall survival, median PFS, and increasing the objective remission rate in patients with HER2-positive or low-expression breast cancer; however, the treatment is associated with notable adverse events, and physicians should be alert to the occurrence of serious adverse events when using this drug.

## 1. Introduction

In 2020, 2 million new cases of breast cancer were reported to reach 2.26 million worldwide, surpassing lung cancer (2.2 million) as the number 1 cancer.^[1]^ Although breast cancer patients have experienced significant clinical benefits as treatment options have been refined, the OS ofpatients with metastatic breast cancer is only about 22 months.^[2]^Overexpression of the HER2 gene fragment is present in 15% to 20% of metastatic breast cancer patients and is associated with multiple organ metastases and poor prognosis.^[3]^Therefore, drug therapy targeting HER2 receptor blockade is essential for these patients.Trastuzumab was approved by Food and Drug Administration in 1998 for the treatment of HER2 + breast cancer.^[4]^ However, some patients treated with trastuzumab still experience disease progression,^[5]^ which forces the physicians to urgently explore new therapeutic directions based on targeted therapy. Trastuzumab emtansine (T-DM1) was approved for marketing applications in 2013 for the treatment of HER2 + breast cancer patients,^[6]^ but its low membrane permeability prevented the cytotoxic drug from reaching the neighboring HER2 low-expressing cells and exhibited bystander killing,^[5]^ which prompted the development of next-generation antibody-drug conjugate (ADC) trastuzumab deruxtecan (T-DXd).

T-DXd is a HER2-targeting ADC consisting of trastuzumab, a cleavable peptidyl linker and topoisomerase I inhibitor (DXd).^[7]^ DXd is a camptothecin derivative, that exhibits strong topoisomerase I inhibition by stably binding to topoisomerase I and DNA complexes mainly through hydrogen bonding, causing inhibition of DNA replication, cell cycle arrest and apoptosis in tumor cells.^[8]^T-DXd also improves the plasma stability of the drug during its antitumor process through a novel peptidyl linker, thus reducing the side effects of cytotoxic drug exposure in body circulation.^[9]^ Its main mechanism of action is that it is cleaved by lysosomal enzymes, such as histones B and L, which are overexpressed in tumor cells, upon entering the targeted cancer cells. In addition, the high drug-to-antibody ratio of T-DXd can deliver higher payload concentrations to cancer cells, giving it more potent antitumor activity than other ADCs.^[10]^ T-DM1 therapy is an option for patients with metastatic HER2-positive breast cancer. At present, the clinical efficacy of T-DM1 has been preliminarily confirmed in different regions, but its specific efficacy has been shown to differ in different studies.^[11]^

Although a Meta-analysis published in 2023 systematically evaluated the efficacy and safety of T-DXd, its data were incomplete due to the pending study deadline, and its results need to be further evaluated.We summarized the updated data on the basis of available evidence by Meta-analysis to assess the efficacy and safety of comparative T-DXd treatment for advanced metastatic HER2 + or HER2 low-expression breast cancer, with the aim of providing a decision-making basis for clinical practice.We have written this paper in a more standardized manner based on the PRISMA^[12]^ statement and the AMSTAR2^[13]^ scale incorporating the latest high-quality evidence and improving methodological treatments.

## 2. Materials and methods

This study was registered with the International Prospective Register of Systematic Reviews (PROSPERO Registration ID: CRD42025632205) and rigorously written following the standards of PRISMA (2020).^[12]^

### 2.1. Data sources and search strategy

An electronic search was performed using the PubMed, EMBASE, and Web of Science databases in July 2025 using a combination of subject words and free words for relevant studies that would be suitable for inclusion in this meta-analysis. Search terms included “breast cancer,” “breast tumor,” “ T-DXd,” “DS-8201a” and “randomized controlled trial,” which were linked with Boolean operators “AND” and “OR.” The first 2 authors determined the final search strategy after several pre-searches, where the disagreement were decided by the third author. Taking PubMed as an example, the specific search strategy is summarized in Table S1, Supplemental Digital Content, https://links.lww.com/MD/Q651.

### 2.2. Inclusion and exclusion criteria

The inclusion criteria were specified using the PICOS model as follows:

1. Population: patients pathologically diagnosed with HER2 + or HER2 low-expressing breast cancer, irrespective of race,

country and the clinical stage.

1. Intervention: One group of patients received T- DXd; the other group received the physician’s choice of treatment such as Capecitabine, Eribulin, Gemcitabine, Paclitaxel and Nab-paclitaxel or no treatment regimen.
2. Comparators: Drug efficacy and safety of the T-DXd and control groups were compared; there were no between-group comparisons in the single-arm trial.
3. Endpoints: median progression-free survival (mPFS);median overall survival(mOS); objective response rate (ORR); incidence of adverse events of any grade and grade ≥ 3.
4. Study design: randomized controlled trials (RCTs), nonrandomized trials, and single-arm studies.

Studies that met the following criteria were excluded.

1. Other tumors were also observed in the study population.
2. If the same study appeared in multiple databases, or literature with updated data owing to different years, only the literature with the most recent and complete information was selected.
3. Literature with unclear or incomplete data or where conversion was not possible.
4. Literature in the form of reviews, editorials, laboratory articles, meta-analyses, observational studies, letters, case reports and reviews.
5. Studies in languages other than English.

### 2.3. Data extraction and literature quality evaluation

Double-blind literature screening, data extraction and quality assessment were conducted by the first and second authors. The results were checked again and harmonized after mutual exchange, and disputes were adjudicated by the third author. The extracted literature information mainly included the study time, clinical trial registration number, baseline characteristics of the study population, interventions, and outcome indicators.

The quality of the included single-arm trials was assessed according to the MINORS scale,^[14]^ and the quality of the included randomized controlled studies was assessed according to the Cochrane Handbook for Systematic Evaluation 5.1.0. The risk of bias was evaluated according to the criterion of “’low risk’ is yes; ‘high risk’ is no; ’unclear’ is lack of relevant information or uncertainty about bias.”^[15]^

### 2.4. Statistical analysis

This study was performed using Stata 14.0 software. Median PFS and mOS effects were analyzed using hazard ratio and 95%CI, and subgroup analyses were carried out based on the baseline characteristics of the patients. ORR and adverse events were analyzed using RR and 95% CI. Heterogeneity was assessed according to the results of Cochran Q and I² tests. Cochran Q statistic of *P* < .1 or I^2^ > 50% indicated high heterogeneity, in which case a random effects model was used; otherwise, a fixed effects model was used. Statistical significance was set at *P* < .05 was considered statistically significant.^[16]^Sensitivity analysis was used to evaluate the impact of each study on the overall hazard ratio. Egger test was used to assess the trial error.

## 3. Results

### 3.1. Results of literature screening and basic information on included studies

We retrieved 3402 relevant literatures. After multiple screenings, we finally included 9 papers (seven studies).^[17–25]^ The long period of time of these 7 studies and the need for researchers to update the progress of the studies resulted in different results of the same study being published in different literatures; therefore 9 papers were included to cover the most complete results of these 7 studies. Finally, We included 2995 patients from the 6 studies.

### 3.2. Basic characteristics of the included studies

The 7 studies covered a total of 2995 patients, with 3 single-arm trials and 4 RCTs. There were 2555 patients in RCTs and 440 patients in single-arm trials. All 4 RCTs were large-sample, multicenter studies. The trial DESTINY-Breast03 compared the treatment effects of T-DXd and T-DM1, while the other 2 RCTs (DESTINY-Breast02,DESTINY-Breast04 and DESTINY-Breast 06) compared T-DXd treatment with previous standard chemotherapy regimens. The basic characteristics of the included studies are presented specifically in Table 1.

**Table 1:** General characteristics of studies included in the meta-analysis.

Study	Year of study	NCT number	Phase	Study Design	Eligible patients	ECOG (T/C,n)		Patients, n (T/ C)	Treatment (single-arm trials)	Treatment(randomized controlled trials)		Endpoints
						0	1or2			T	C	
DS8201-A-J101^[17]^	2015.09–2024.01	NCT02564900	Ⅰ	A multicenter/open-Label/multi-dose and single-group trial	HER2 low expression metastatic breast cancer patients resistant to standard therapy	72	43	115	5.4 or 6.4 mg/kg T -DXd	–	–	①②③④⑤
DAISY^[18]^	2019.10–2023.12	NCT04132960	Ⅱ	A Multicenter/open-Label and single-group trial	Cohort 1: HER2 over expression breast cancer patients (HER2 IHC3 + or HER2 IHC2 +/ ISH+)	21	47	68	5.4 mg/kg T-DXd	–	–	①②③④⑤
					Cohort 2: HER2 low expression breast cancer patients (IHC1 + or IHC2 +/ ISH-)	33	40	73	5.4mg/kg T-DXd	–	–	①②③④⑤
DESTINY-Breast01^[19,20]^	2017.08–2024.09	NCT03248492	Ⅱ	A multicenter/open-Label and single-group trial	HER2 + metastatic breast cancer patients previously treated with T-DM1	102	82	184	5.4mg/kg T-DXd	–	–	①②③④⑤
DESTINY-Breast02^[21]^	2018.04–2024.09	NCT03523585	Ⅲ	A multicenter/open-Label and randomized controlled trial	HER2 + metastatic breast cancer patients previously treated with T-DM1	228/121	178/81	608 (406:202)	–	5.4 mg/ kg T-DXd	Capecitabine + trastuzumab or Capecitabine + Lapatinib	①②③④⑤
DESTINY-Breast03^[22,23]^	2018.04–2024.06	NCT03529110	Ⅲ	A multicenter/open-Label and randomized controlled trial	HER2 + metastatic breast cancer patients previously treated with trastuzumab and paclitaxel	154/175	106/87	524 (261:263)	–	5.4 mg/ kg T-DXd	T-DM 13.6 mg/kg	①②③④⑤
DESTINY-Breast04^[24]^	2018.11–2024.04	NCT03734029	Ⅲ	A multicenter/ open-Label and randomized controlled trial	HER2 low expression metastatic breast cancer patient resistant to standard therapy	187/95	144/68	557 (373:184)	–	5.4 mg/ kg T-DXd	Capecitabine, Eribulin, Gemcitabine, Paclitaxel or Nab-Paclitaxel	①②③④⑤
DESTINY-Breast06^[25]^	2020.08–2024.03	NCT04494425	Ⅲ	A multicenter/ Open-Label and randomized controlled trial	Hormone receptor- positive, HER2 low-expressing or HER2- ultra-low-expressing metastatic breast cancer	252/257	179/164	866 (436:430)	–	5.4 mg/ kg T-DXd	Capecitabine, Nab-paclitaxel, or Paclitaxel	①②③④⑤

①median progression-free survival; ②median overall survival; ③objective response rate; ④incidence of adverse events at any level; ⑤incidence of adverse events above level III. HER2 low-expressing: score of 1 + or 2 + on immunohistochemical [IHC] analysis and negative results on in situ hybridization; HER2 ultra-low-expressing: IHC 0 with membrane staining.

### 3.3. Quality assessment of included studies

Three single-arm trials of the 7 studies were quality assessed using the criteria related to the MINORS scale (Table S2, Supplemental Digital Content, https://links.lww.com/MD/Q651), and 4 RCTs were quality tested using Cochrane Handbook for Systematic Evaluation 5.1.0. Seven studies assessed a low risk of bias. Except for DS8201-A-J1017, DAISY, and DESTINY-Breast01, which lacked OS results, the endpoints of the remaining studies were completely reported (Table 2; Table S3, Supplemental Digital Content, https://links.lww.com/MD/Q651).

**Table 2:** Risk of bias assessment for RCTs.

Study	Random sequence	Assignment hiding	Participant and practitioner blindness	Outcome assessment blinding	Incomplete ending data	Selective reporting	Other biases
DESTINY-Breast02	Low risk	Unclear	Low risk	Low risk	Low risk	Low risk	Unclear
DESTINY-Breast03	Low risk	Unclear	Low risk	Low risk	Low risk	Low risk	Unclear
DESTINY-Breast04	Low risk	Unclear	Low risk	Low risk	Low risk	Low risk	Unclear
DESTINY-Breast06	Low risk	Unclear	Low risk	Low risk	Low risk	Low risk	Unclear

RCTs = randomized controlled trials.

### 3.4. Meta-Analysis

#### 3.4.1. Median progression-free survival

**Figure 1.**
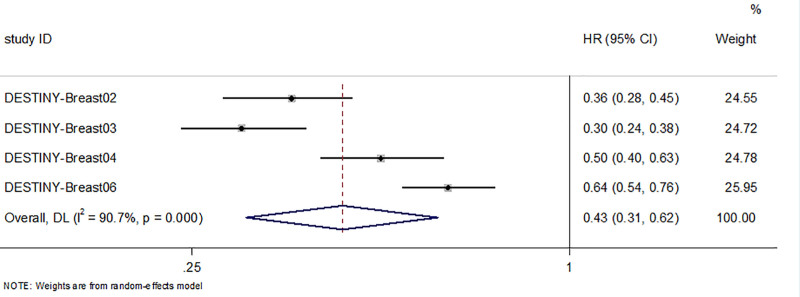
Forest plot of mPFS in RCTs. RCTs = randomized controlled trials.

Four randomized controlled studies included 2555 patients, of which 1476 patients were included in the T-DXd group and 1079 patients in the control group. The test of heterogeneity resulted in I^2^ = 90.7%; therefore a random effects model was used, which showed that patients in the T-DXd group achieved a longer median PFS than patients in the control group (HR = 0.43; 95% CI:0.31–0.62, *P* < .05, Figure 1). On the other hand, the 3 single-arm studies showed that the median PFS of the 375 patients included ranged from 6.7 to 19.4 months(Table 3).

**Table 3:** ORR and mPFS in single-arm trials.

Study	Year of study	NO. of Patients	Median PFS (95% CI) (Mo)	ORR (95% CI) (%)
DS8201-A-J101	2015.09–2024.01	51	13.7 (8.5–19.6)	59.5 (49.7–68.7)
DAISY Cohort 1	2019.10–2023.12	68	11.1 (8.5–14.4)	70.6 (58.3–81.0)
DAISY Cohort 2		72	6.7 (4.4–8.3)	37.5 (26.4–49.7)
DESTINY-Breast01	2017.08–2024.09	184	19.4 (14.1–25.0)	62.0 (54.5–69.0)

mPFS = median progression-free survival, ORR = objective response rate.

#### 3.4.2. Median overall survival

**Figure 2.**
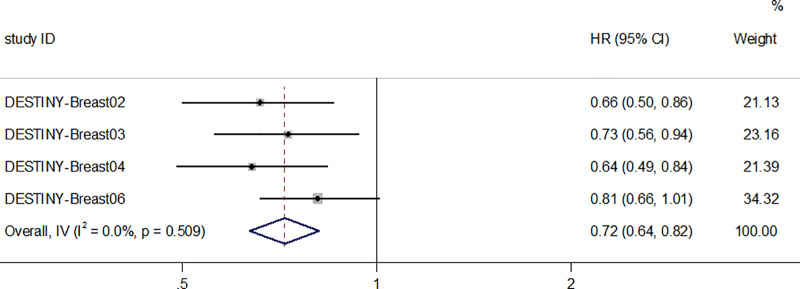
Forest plot of mOS in RCTs. mOS = median overall survival, RCTs = randomized controlled trials.

Four studies reported relevant mOS data involving 2555 patients, and all 4 studies were randomized controlled studies.A total of 1476 patients were included in the T-DXd group and 1079 patients in the control group, and the median overall survival of T-DXd patients was longer than that of control patients (HR = 0.72; 95% CI:0.64–0.82, p<0.05, Figure 2). OS-related efficacy analyses were not performed in this study because the data from the single-arm trials were not mature or applicable.

#### 3.4.3. Objective response rate

**Figure 3.**
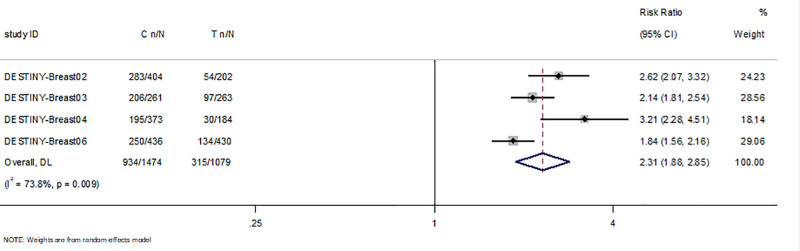
Forest plot of ORR in RCTs. RCTs = randomized controlled trials.

**Figure 4.**
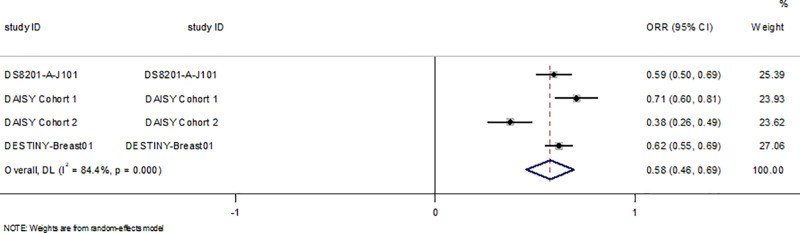
Forest plot of ORR in single-arm trials. ORR = objective response rate.

ORR data were available for all studies.We included 1474 patients treated with T-DXd and 1079 patients treated with other therapies in RCTs.Objective response was achieved in 934 of the 1474 patients treated with T-DXd, compared with 315 of the 1079 patients in the control group. The results showed that T-DXd significantly improved ORR in the HER2 + or low-expression breast cancer population compared to the control group (RR = 2.31; 95%CI: 1.88–2.85, P<0.05, Fig. 3). Objective responses were achieved in 255 of 435 patients in 3 single-arm trials, with objective response rates ranging from 37.5% to 70.6% across studies. We found that the ORR of patients with breast cancer treated with T-DXd was 58% (95%CI:46,69)(Fig. 4 and Table 3).

#### 3.4.4. Adverse events

A total of 440 patients were reported to have adverse events in the 4 single-arm studies, and we performed a pooled prevalence analysis of AEs from each single-arm trial. The results showed that nausea had the highest prevalence of 36% (CI 95%: 4, 14), followed by fatigue (25%), vomiting (24%), alopecia (19%) and anemia (18%) among any grade of adverse events. The pooled prevalence of grade ≥ 3 adverse events was generally low, with the highest prevalence of anemia and neutropenia at 11% (CI 95%: 4, 19) and 8% (CI 95%: 4, 14), respectively (Table 4).

**Table 4:** Meta-analysis of adverse events in the single-arm study.

Adverse events	DS8201-A-J101	DAISY Cohort 1	DAISY cohort 2	DESTINY-Breast01	Effects model	Overall (95% CI)	*P*	*I* ^2^
Any grade								
Anemia	0.39 (0.30–0.49)	0.04 (0.01–0.12)	0.05 (0.02–0.13)	0.30 (0.23–0.37)	Random	0.18 (0.04–0.36)	0.00	94.84
Fatigue	0.44 (0.35–0.54)	0.09 (0.03–0.18)	0.07 (0.02–0.15)	0.49 (0.42–0.57)	Random	0.25 (0.07–0.50)	0.00	96.47
Nausea	0.79 (0.71–0.86)	0.01 (0.00–0.08)	0.07 (0.02–0.15)	0.78 (0.71–0.84)	Random	0.36 (0.03–0.82)	0.01	98.98
Vomiting	0.52 (0.43–0.62)	0.03 (0.00–0.10)	0.10 (0.04–0.19)	0.46 (0.38–0.53)	Random	0.24 (0.05–0.52)	0.00	97.09
Constipation	0.37 (0.28–0.46)	0.00 (0.00–0.05)	0.01 (0.00–0.07)	0.36 (0.29–0.43)	Random	0.13 (0.00–039)	0.03	97.39
Diarrhea	0.37 (0.29–0.47)	0.00 (0.00–0.05)	0.04 (0.01–0.12)	0.29 (0.23–0.36)	Random	0.13 (0.01–0.36)	0.01	96.62
Neutropenia	0.47 (0.38–0.56)	0.01 (0.02–0.14)	0.04 (0.01–0.12)	0.35 (0.28–0.42)	Random	0.16 (0.04–0.34)	0.00	94.37
Alopecia	0.47 (0.38–0.56)	0.01 (0.00–0.08)	0.01 (0.00–0.07)	0.48 (0.41–0.56)	Random	0.19 (0.01–0.51)	0.02	97.98
Interstitial lung disease	0.05 (0.02–0.11)	0.01 (0.00–0.08)	0.00 (0.00–0.05)	0.14 (0.09–0.19)	Random	0.04 (0.00–0.12)	0.03	88.62
Grade ≥ 3								
Anemia	0.17 (0.10–0.25)	0.03 (0.00–0.10)	0.05 (0.02–0.13)	0.09 (0.05–0.14)	Random	0.08 (0.04–0.14)	0.00	72.41
Fatigue	0.04 (0.01–0.10)	0.01 (0.00–0.08)	0.07 (0.02–0.15)	0.06 (0.03–0.10)	fixed	0.06 (0.04–0.08)	0.00	0.00
Nausea	0.03 (0.01–0.09)	0.03 (0.01–0.10)	0.07 (0.02–0.15)	0.08 (0.04–012)	fixed	0.05 (0.03–0.07)	0.00	0.00
Vomiting	0.04 (0.01–0.10)	0.01 (0.00–0.08)	0.08 (0.03–0.17)	0.04 (0.02–0.08)	fixed	0.04 (0.03–0.06)	0.00	0.00
Constipation	0.01 (0.00–0.05)	0.00 (0.00–0.05)	0.00 (0.00–0.05)	0.00 (0.00–0.05)	fixed	0.00 (0.00–0.01)	0.15	0.00
Diarrhea	0.02 (0.00–0.06)	0.00 (0.00–0.05)	0.04 (0.01–0.12)	0.03 (0.01–0.06)	fixed	0.02 (0.01–0.04)	0.00	0.00
Neutropenia	0.14 (0.08–0.22)	0.06 (0.02–0.14)	0.04 (0.01–0.12)	0.21 (0.15–0.27)	Random	0.11 (0.04–0.19)	0.00	83.51
Alopecia	0.00 (0.00–0.03)	0.01 (0.00–0.08)	0.01 (0.00–0.07)	0.401 (0.00–0.03)	fixed	0.00 (0.00–0.02)	0.06	0.00
Interstitial lung disease	0.01 (0.00–0.05)	0.01 (0.00–0.08)	0.00 (0.00–0.05)	0.01 (0.00–0.03)	fixed	0.01 (0.00–0.02)	0.05	0.00

We performed a meta-analysis of the incidence of common adverse events in randomized controlled studies including 2555 patients. The results showed that nausea and fatigue were the most common adverse events of any grade, and their incidence was 53.33% and 36.92%, respectively, which was similar to the results of the single-arm trial. Patients in the T-DXd group had a significantly higher incidence of fatigue, anemia, nausea, vomiting, alopecia, constipation, and interstitial lung disease compared to the control patients, whereas no significant differences were confirmed between the 2 groups in terms of diarrhea and neutropenia.The overall incidence of grade ≥ 3 adverse events was low, with anemia and neutropenia being the most common adverse events (7.05% and 13.07%, respectively), which was consistent with the results of the single-arm trial.The incidence of anemia, fatigue, nausea, interstitial lung disease, diarrhea, vomiting and constipation was significantly higher in patients treated with T-DXd than in control patients, whereas the differences in neutropenia and alopecia between the 2 groups were not significant. Notably, the incidence of interstitial lung disease cited as an adverse event of special concern was 7.14% for any grade and 0.92% for grade ≥ 3 (Table 5).

**Table 5:** Meta-analysis of adverse events in randomized controlled trials.

Adverse events	T-DXd Group (n/N)	control Group (n/N)	Total incidence(%)	Heterogeneity		Effects model	RR (95% CI)	*P*
				*I*^2^(%)	*P*			
Any grade								
Anemia	455/1466	198/1045	26.01	20.40	.288	Fixed	1.65 (1.42–1.92)	.000
Fatigue	606/1466	321/1045	36.92	11.20	.337	Fixed	1.32 (1.18–1.47)	.000
Nausea	1048/1466	291/1045	53.33	71.30	.015	Random	2.53 (2.08–3.07)	.000
Vomiting	594/1466	160/1045	30.03	0.00	.616	Fixed	2.82 (2.42–3.29)	.000
Constipation	317/1032	94/628	24.76	64.40	.06	Random	2.16 (1.49–3.13)	.000
Diarrhea	378/1466	251/1045	25.05	96.20	.00	Random	1.25 (0.59–2.65)	.561
Neutropenia	444/1466	247/1045	27.52	95.10	.00	Random	1.55 (0.81–2.92)	.182
Alopecia	589/1466	154/1045	29.59	95.40	.00	Random	3.85 (1.66–8.92)	.002
Interstitial lung disease	175/1466	11/1045	7.41	53.50	.091	Random	13.73 (4.31–43.68)	.000
Grade ≥ 3								
Anemia	111/1466	41/1045	6.05	0.00	.613	Fixed	1.92 (1.34–2.74)	.000
Fatigue	75/1466	17/1045	3.66	38.20	.183	Fixed	2.89 (1.73–4.84)	.000
Nausea	69/1466	7/1045	3.03	37.20	.189	Fixed	6.09 (2.87–12.92)	.000
Vomiting	44/1466	3/1045	1.87	0.00	.488	Fixed	10.01 (3.29–30.46)	.000
Constipation	32/1032	6/628	2.29	0.00	–	Fixed	2.57 (1.09–6.05)	.030
Diarrhea	26/1466	29/1045	2.19	0.00	.446	Fixed	0.58 (0.35–0.98)	.040
Neutropenia	225/1466	151/1045	14.97	96.1	.000	Random	1.72 (0.55–5.40)	.353
Alopecia	2/1466	1/1045	0.11	0.00	.564	Fixed	1.63 (0.13–20.96)	.709
Interstitial lung disease	21/1466	2/1045	0.92	0.00	.550	Fixed	6.93 (1.60–29.91)	.009

n, number of persons involved in incidents; N, total number.

#### 3.4.5. Subgroup analysis

We performed a subgroup analysis of the mPFS of the eligible RCTs in terms of hormone receptor status, previous lines of therapy, brain metastases, and visceral metastases.The results showed that the mPFS of patients in the T-DXd group was significantly longer than that of patients in the control group regardless of whether the patients were positive or negative for hormone receptors, the presence of brain metastases, the presence of <3 previous systemic therapies and the presence of baseline visceral disease (Table 6).

**Table 6:** Subgroup analysis of mPFS.

Subgroup	Number of tests	Heterogeneity		HR (95% CI)	*P*
		I^2^	*P*		
Hormone receptor-positive	3	13.40%	.315	0.45 (0.31–0.57)	<.001
Hormone receptor negative	3	0.00%	.585	0.34 (0.27–0.43)	<.001
Baseline brain metastases (yes)	2	0.00%	.583	0.31 (0.21–0.47)	<.001
Baseline brain metastases (no)	2	0.00%	.736	0.37 (0.30–0.46)	<.001
Baseline visceral disease (yes)	2	0.00%	.643	0.35 (0.29–0.42)	<.001
Baseline visceral disease (no)	2	0.00%	.891	0.38 (0.26–0.55)	<.001
Previous lines of systemic therapy<3	2	0.00%	.623	0.37 (0.30–0.46)	<.001
Previous lines of systemic therapy ≥3	2	17.40%	.271	0.37 (0.28–0.48)	<.001

mPFS = median progression-free survival.

In addition, we took into account the significant differences in biology, prognosis, and treatment between the 2 groups of HER2-positive and HER2-low expression treated by T-DXd. Therefore, further subgroup analyses of the included studies according to HER2 expression were done to report the specific meta-results of the 2 subgroups more clearly. The results showed that the mPFS, mOS and ORR of T-DXd treatment for both HER2-positive and low-expression groups were significantly better than that of the control medication (Table 7).

**Table 7:** Subgroup analysis on HER2 typing.

End points	HER2 typing	Number of tests	Heterogeneity		HR (95% CI)	*P*
			*I* ^2^	*P*		
mPFS	HER2-positive	2	14.6%	.279	0.33 (0.28,0.39)	<.05
	HER2 low expression	2	65.5%	.089	0.57 (0.45,0.73)	<.05
mOS	HER2-positive	2	0.00%	.598	0.70 (0.58,0.84)	<.05
	HER2 low expression	2	44.7%	.179	0.74 (0.63,0.87)	<.05
ORR	HER2-positive	2	48.8%	.162	2.35 (2.04,2.70)	<.05
	HER2 low expression	2	87.9%	.004	2.38 (1.38,4.09)	<.05

mOS = median overall survival, mPFS = median progression-free survival, ORRs = objective response rates.

### 3.5. Publication bias analysis

We plotted funnel plots for mOS, mPFS and ORR in RCTs and single-arm trials separately to test for publication bias(Fig. S1, Supplemental Digital Content, https://links.lww.com/MD/Q651). As a result, we did not find significant asymmetry, which indicated no significant publication bias.The results of Egger test further supported this result (RCTs: mPFS *P* = .146; mOS *P* = .057; ORR *P* = .067; single-arm trials: PFS *P* = .934; ORR *P* = .634) (Table S4, Supplemental Digital Content, https://links.lww.com/MD/Q651).

### 3.6. Sensitivity analysis

**Figure 5.**
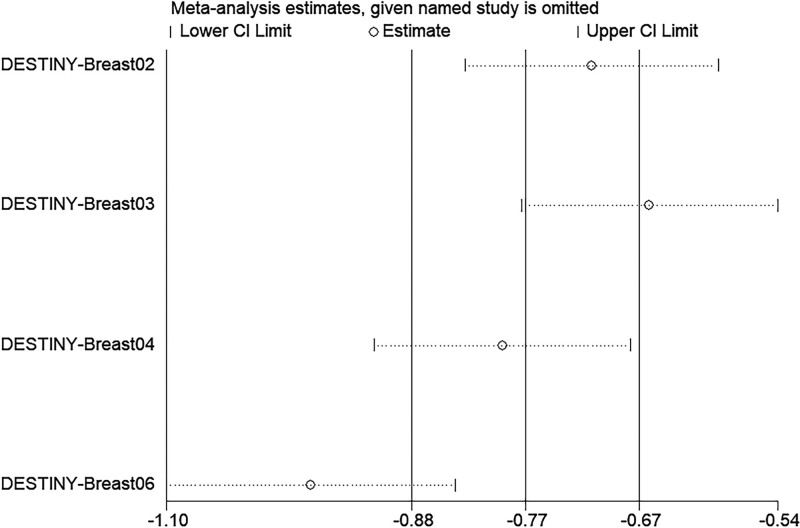
Leave-one-out sensitivity analysis of mPFS. mPFS = median progression-free survival.

We found that some of the studies with large effect sizes in the Meta-analysis results had some possibility of dispersion, so we conducted a sensitivity analysis for this study.After the results of this study were excluded one by one, the combined effect sizes did not change statistically significant changes, which indicated that the results of this study were relatively robust (Fig. 5).

## 4. Discussion

Currently, targeted therapy is the standard treatment option for patients with HER2 + breast cancer. However, as it has been widely used, resistance to targeted drugs has gradually emerged, which has resulted in reduced benefits for breast cancer patients and forced physicians to seek more effective and rational treatment options such as trastuzumab emtansine(T-DM1) and trastuzumab deruxtecan,which have been successively approved by the Food and Drug Administration for breast cancer treatment since 2013.^[6]^ ADC has also become a hot research topic in recent years.A study^[19]^ using T-DXd at a dose of 5.4 mg/kg for the treatment of HER2 + breast cancer found that T-DXd prolonged mPFS and mOS, while a phase III clinical study, called DESTINY-Breast04, found that T-DXd prolonged the mPFS of metastatic breast cancer with HER2 low expression to 9.9 months, confirming its significant effect in prolonging PFS.^[24]^

The results of our study further confirmed the benefit of T-DXd in prolonging mPFS in patients with metastatic HER2 + or HER2 low expression. Notably, we observed a high degree of heterogeneity in the mPFS forest plots and for this reason systematically excluded 1 study in each case. As a result, no significant reduction in heterogeneity was obtained after excluding either study, and we then systematically explored the differences between the included studies to investigate the reasons for the high heterogeneity. Analysis of the reasons for this revealed that 2 studies were designed in populations with positive HER2 expression, whereas the other 2 were designed in populations with low and ultra-low HER2 expression. Although both HER2-positive and HER2-low expression are suitable for T-DXd, HER2-positive breast cancers may benefit more significantly due to the high level of HER2 protein expression, with which T-DXd binds more efficiently to exert its targeting effect. Accordingly, we performed subgroup analyses according to the HER2 typing of the study population, and the issue of high heterogeneity was addressed. And subgroup analysis showed that the HER2 positive expression group showed a more significant and stable benefit in mPFS and mOS than the low expression group. The ORR multiplicity of enhancement was close in both groups (2.35 vs 2.38), but the confidence interval was narrower in the positive expression group (95% CI width 0.66 vs 2.71), and the heterogeneity was significantly lower (I²=48.8% vs 87.9%), suggesting that the efficacy of HER2 positive expression was more reliable. The above suggests that HER2 positive expression is a clear therapeutic target, while the clinical value of low expression still needs to be validated by larger studies. In addition, we also found that T-DXd resulted in longer mOS, which is consistent with the results of a meta-analysis conducted by Dowling.^[26]^ However, data related to OS are currently awaiting further maturation owing to the study timeframe constraints. In other exploratory analyses of efficacy, we found that T-DXd significantly improved ORR in the ITT population. The DESTINY-Breast02 study, which enrolled the largest number of patients, demonstrated that patients with breast cancer on trastuzumab deruxtecan had a higher ORR(70.0% vs 29.0%, *P* < .05) and longer mPFS (17.8 vs 6.9, *P* < .001) than their physician’s choice of treatment regimen, which is consistent with the results of this study. This suggests that the trastuzumab deruxtecan is efficacious in patients with HER2 + breast cancer.

Our subgroup analysis revealed that the mPFS of patients treated with T-DXd was significantly prolonged regardless of the presence of brain metastases or baseline visceral disease, hormone receptor status, and the presence of less than 3 previous systemic therapies, suggesting that T-DXd has an advantage in the terms of therapeutic spectrum. Moreover, it was found^[27]^ that approximately 50% of HER2 + metastatic breast cancers develop brain metastases and have a worse prognosis than patients without brain metastases. A clinical trial called TUXEDO-1^[28]^ found that endpoints related to patients with brain metastases from HER2 + breast cancer receiving T-DXd met the predicted outcomes and no new adverse events were observed, which is consistent with the results of the brain metastasis subgroup analyses in our study, confirming the high extracranial activity of trastuzumab deruxtecan in HER2 + breast cancer. In addition, considering the significant biological, prognostic and therapeutic differences between the 2 cohorts of HER2 + and HER2 low expression populations treated with T-Dxd, we also performed a subgroup analysis of HER2 typing. The results found that T-DXd treatment of both the HER2 positive expression and low expression groups had significantly better mPFS, mOS and ORR than the control group, which also confirmed the broad advantages of its targeted HER2 therapy, filling the gap of the low expression population that could not be covered by the traditional HER2-targeted therapies, and making it an important choice for such patients. In sensitivity analyses, we found that while the treatment effects of the DESTINY-Breast series of studies were statistically significant and robust, and the combined results support the clinical value of the therapy, DESTINY-Breast06 showed the strongest effect (lowest estimate) and widest confidence interval, suggesting a reduced survival benefit or event rate in the treatment group. We explored the reasons for this and found that the study’s inclusion population was predominantly HER2 low-expressing or HER2-ultra-low-expressing metastatic breast cancer. Although the study showed an improvement in the scores associated with patients with ultra-low HER2 expression, the overall results of the study were also somewhat influenced by the survival benefit due to targeted binding limitations. The ongoing DESTINY-Breast08 trial mainly enrolled breast cancer patients with low HER2 expression, ER or PR-positive (ER or PR ≥ 1%) or ER and PR-negative (ER and PR < 1%), aiming at exploring the safety, tolerability, pharmacokinetics, and antitumor activity of T-DXd in combination with other anticancer drugs, and the results of which are also awaiting maturation.^[29]^ Ongoing studies in people with low HER2 expression also include clinical trials in combination with anastrozole and an EXH 1/2 inhibitor called vale-metostat. The above trials will confirm the feasibility of T-DXd as a first-line treatment for HR-negative HER2 low-expressing breast cancer in the future.

However, the increased efficacy of T-DXd is inevitably accompanied by adverse events. In terms of adverse events, trastuzumab deruxtecan increased the incidence of fatigue, anemia, nausea, vomiting, constipation, and interstitial lung disease. Interstitial lung disease has been examined in several studies as a life-threatening complication. As with other anticancer drugs, the mechanisms of underlying T-DXd-associated interstitial lung disease are unknown. The current hypothesis suggests that interstitial lung disease may be related to target-dependent or nondependent uptake and catabolism of ADCs, in addition to bystander killing of payloads released from cells after ADC catabolism.^[30]^ And it was found that T-DXd was mainly distributed in alveolar macrophages, based on which a noncancer target mechanism has been proposed.^[31]^ The sustained treatment of T-DXd is hampered by interstitial lung disease, and ILD can be fatal if not controlled in time.^[32]^ Therefore, physicians should closely monitor the occurrence of interstitial lung disease during the use of T-DXd. Although alopecia and neutropenia occurred at higher rates, we did not find them to be significantly different from the control group, suggesting that T-DXd did not significantly increase the risk of occurrence in these areas.Although T-DXd is a good option, its use must be balanced with potentially serious side effects, which requires careful selection of the applicable patient population and prudent monitoring by clinicians during treatment.

We have summarized the updated data based on what was already available. The literature included in this study was of high quality, but there were some limitations: 1) Data on overall survival in the included trials were not yet mature enough to be analyzed; 2) The included studies may have a potential risk of bias.They contained many single-arm trials. The RCTs were conducted.. And all of the trials had financial support from drug companies; 3) The T-DXd doses in the included studies included 6.4 mg/kg or 5.4 mg/kg, with no standardized dosage criteria; 4) We included fewer than 10 studies and funnel plot testing did not reveal significant publication bias. However, it is not yet possible to accurately determine whether the results of the publication bias are credible.

In summary, we systematically evaluated the efficacy and safety of Trastuzumab deruxtecan in patients with HER2 + or HER2 low-expressing breast cancer, and also explained the reasons for the increased incidence of some adverse events, which provides an evidence-based basis for the monitoring of the drug use. It is suggested that large-sample multicenter clinical studies could be conducted from the perspectives of adverse effects and dosage, and further validated in terms of cost-effectiveness.

## Acknowledgments

The author wishes to express their gratitude to the team for their valuable contributions, help, and insightful discussions throughout the project.

## Author contributions

**Conceptualization:** Yujing Mu, Yingyi Fan, Xiaohua Pei.

**Data curation:** Yujing Mu, Jianrong Li, Yingyi Fan.

**Formal analysis:** Yujing Mu, Jianrong Li.

**Funding acquisition:** Xiaohua Pei.

**Methodology:** Yujing Mu.

**Supervision:** Xiaohua Pei.

**Validation:** Xiaohua Pei.

**Writing – original draft:** Yujing Mu, Yingyi Fan.

**Writing – review & editing:** Yujing Mu, Xiaohua Pei.

## Supplementary Material


