# Supplementary material for: Efficacy and safety of trastuzumab deruxtecan for metastatic HER2+ and HER2-low breast cancer: A systematic review and meta-analysis
Source: Medicine (Baltimore). 2025 Nov 14;104(46):e45936. doi: 10.1097/MD.0000000000045936 (PMC12622611; doi:10.1097/MD.0000000000045936)

Supplemental File 1. PubMed search strategy.

#1 breast cancer [MeSH Terms]

#2 ((breast OR mammary) AND (carcinoma OR neoplasm OR tumor OR cancer))

#3 #1 OR #2

#4 Trastuzumab Deruxtecan OR DS-8201 OR Enhertu OR T-DXd

#5 #4 AND #3

#6 ((((randomized controlled trial [pt] OR controlled clinical trial [pt] OR randomized controlled trials [mh] OR random allocation [mh] OR double-blind method [mh] OR single-blind method [mh] OR clinical trial [pt] OR clinical trials [mh] OR (「clinical trial」[tw]) OR ((singl* [tw] OR doubl* [tw] OR trebl* [tw] OR tripl* [tw]) AND(mask* [tw] OR blind* [tw])) OR (placebos [mh] OR placebo* [tw] OR random* [tw] OR research design [mh:noexp])NOT (animals [mh] NOT human [mh])))))

#7 #6 AND #5

Box 1.: PubMed search strategy.

Table S1: Full search strategy

Search Library	Search strategy
Web of Science	#1 TS=(breast OR mammary) AND TS=(carcinoma OR neoplasm OR tumor OR cancer)<br>#2 TS=(DS-8201 OR Enhertu OR T-DXd)<br>#3 #1 AND #2<br>#4 TS= clinical trial* OR TS=research design OR TS=comparative stud* OR TS=evaluation stud* OR TS=controlled trial* OR TS=follow-up stud* OR TS=prospective stud* OR TS=random* OR TS=placebo* OR TS=(single blind*) OR TS=(double blind*)<br>#5 #3 AND #4
Pub Med	#1 breast cancer [MeSH Terms]<br>#2 ((breast OR mammary) AND (carcinoma OR neoplasm OR tumor OR cancer))<br>#3 #1 OR #2<br>#4 DS-8201 OR Enhertu OR T-DXd<br>#5 #3 AND #4<br>#6 ((((randomized controlled trial [pt] OR controlled clinical trial [pt] OR randomized controlled trials [mh] OR random allocation [mh] OR double-blind method [mh] OR single-blind method [mh] OR clinical trial [pt] OR clinical trials [mh] OR (「clinical trial」[tw]) OR ((singl* [tw] OR doubl* [tw] OR trebl* [tw] OR tripl* [tw]) AND(mask* [tw] OR blind* [tw])) OR (placebos [mh] OR placebo* [tw] OR random* [tw] OR research design [mh:noexp])NOT (animals [mh] NOT human [mh])))))<br>#7 #6 AND #5
Embase	#1 ('breast'/exp OR breast) AND ('cancer'/exp OR cancer)<br>#2 'T-DXd':ab,ti OR 'DS-8201':ab,ti OR 'Enhertu':ab,ti<br>#3 #1 AND #2<br>#4 'crossover procedure':de OR 'double-blind procedure':de OR 'randomized controlled trial':de OR 'single-blind procedure':de OR random*:de,ab,ti OR factorial*:de,ab,ti OR crossover*:de,ab,ti OR ((cross NEXT/1 over*):de,ab,ti) OR placebo*:de,ab,ti OR ((doubl* NEAR/1 blind*):de,ab,ti) OR ((singl* NEAR/1 blind*):de,ab,ti) OR assign*:de,ab,ti OR allocat*:de,ab,ti OR volunteer*:de,ab,ti<br>#5 #4 AND #3

Six domains were adapted to assess bias in the studies.The items were evaluated according to the Newcastle-Ottawa scale. And the risk of bias was evaluated by defining “reported and adequate” as “low risk”; “not reported” as “high risk”; and “reported but inadequate” as “unclear”. The Newcastle-Ottawa scale for evaluating the quality of single-arm trials is shown in Supplementary Table 2, and the specific evaluation results are shown in Supplementary Table 3.

Table S2 Quality evaluation criteria for single-arm tests

**Newcastle-Ottawa Adapted to Include Single-Arm Trials**
1. **A clearly stated aim:** the question addressed should be precise and relevant in the light of available literature
2. **Inclusion of consecutive patients**: all patients potentially fit for inclusion (satisfying the criteria for inclusion) have been included in the study during the study period (no exclusion or details about the reasons for exclusion)
3. **Prospective collection of data**: data were collected according to a protocol established before the beginning of the study
4. **Endpoints appropriate to the aim of the study**: unambiguous explanation of the criteria used to evaluate the main outcome which should be in accordance with the question addressed by the study. Also, the endpoints should be assessed on an intention-to-treat basis.
5. **Unbiased assessment of the study endpoint**: blind evaluation of objective endpoints and double-blind evaluation of subjective endpoints. Otherwise the reasons for not blinding should be stated
6. **Follow-up period appropriate to the aim of the study**: the follow-up should be sufficiently long to allow the assessment of the main endpoint and possible adverse events
7. **Loss to follow up less than 5%:** all patients should be included in the follow up. Otherwise, the proportion lost to follow up should not exceed the proportion experiencing the major endpoint
8. **Prospective calculation of the study size**: information of the size of detectable difference of interest with a calculation of 95% confidence interval, according to the expected incidence of the outcome event, and information about the level for statistical significance and estimates of power when comparing the outcomes

Table S3 Risk of bias assessment for included single-arm studies

Study	1*	2*	3*	4*	5*	6*	7*	8*
DS8201-A-J101	low risk	low risk	low risk	low risk	low risk	low risk	low risk	low risk
DAISY Cohort 1	low risk	low risk	low risk	low risk	low risk	unclear	low risk	low risk
DAISY Cohort 2	low risk	low risk	low risk	low risk	low risk	unclear	low risk	low risk
DESTINY-Breast01	low risk	low risk	low risk	low risk	low risk	low risk	low risk	low risk

Abbreviations:*Criteria for evaluating items in Table S2.

Table S4 Begg and Egger publication bias test

Tems	Egger's Test		Begg's Test	
	Tval	Pval	Z	Zp
mPFS in randomized control trials	-0.45	0.729	0.00	1.00
mOS in randomized control trials	-2.81	0.218	0.00	1.00
ORR in randomized control trials	7.42	0.085	1.04	0.296
mPFS in single-arm trials	-0.09	0.934	——	——
mOS in single-arm trials	-0.56	0.634	——	——

Abbreviations: Tval, value of t-statistic;Pval, p-value related to T statistic;Z:Z-statistic; Zp: p-value related to Z statistic

Figure S1. Forest plot on publication bias

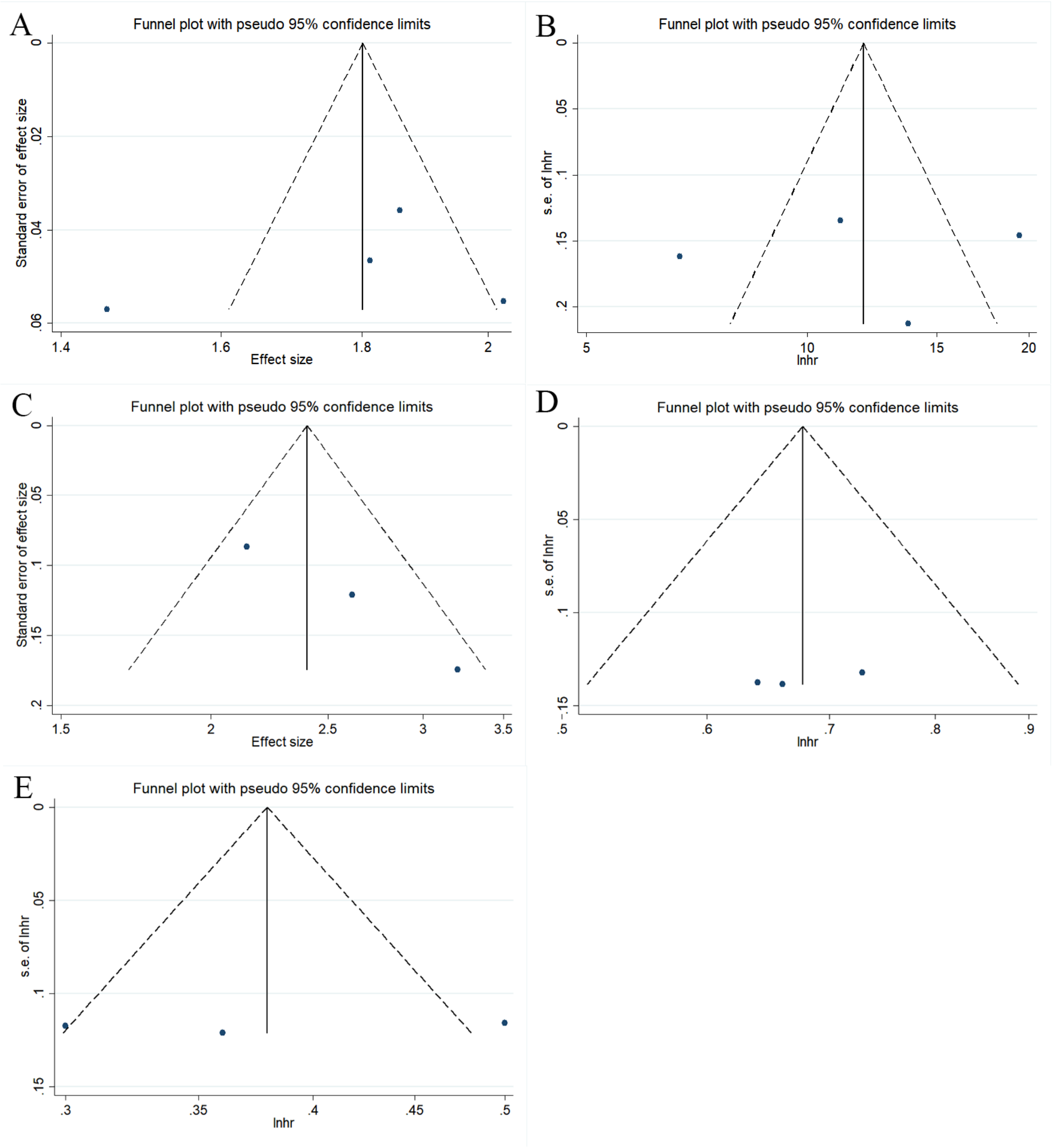

Supplement: Supplementary file 1 [file medi-104-e45936-s001.pdf]
